# Influence of a novel classification of the papilla of Vater on the outcome of needle-knife fistulotomy for biliary cannulation

**DOI:** 10.1186/s12876-021-01735-3

**Published:** 2021-04-01

**Authors:** Jorge Canena, Luís Lopes, João Fernandes, Patrício Costa, Marianna Arvanitakis, Arjun D. Koch, Jan-Werner Poley, Javier Jimenez, Enrique Dominguez-Munõz, Pietro Familiari, Marco J. Bruno, Mário Dinis-Ribeiro

**Affiliations:** 1 Department of Gastroenterology, Professor Doutor Fernando Fonseca Hospital, IC 19, 2720276 Amadora, Portugal; 2 Department of Gastroenterology, Santa Luzia Hospital - Unidade Local de Saúde Alto Minho, Viana do Castelo, Portugal; 3 Department of Gastroenterology, Nova Medical School/Faculty of Medical Sciences, Lisbon, Portugal; 4 University Center of Gastroenterology, Hospital Cuf Tejo, Lisbon, Portugal; 5 Life and Health Sciences Research Institute (ICVS), School of Medicine, University of Minho, Braga, Portugal; 6 ICVS/3B’s - PT Government Associate Laboratory, Braga/Guimarães, Portugal; 7 Cintesis – Center for Health Technology and Services Research, Porto, Portugal; 8 Department of Gastroenterology, Hepatology and Digestive Oncology, Erasme University Hospital Université Libre de Bruxelles, Brussels, Belgium; 9 Department of Gastroenterology and Hepatology, Erasmus MC Cancer Institute, University Medical Center, Rotterdam, The Netherlands; 10 Endoscopy Unit. Hospital Marques de Valdecilla, Santander, Spain; 11 Gastroenterology Department, University Hospital of Santiago de Compostela, Santiago de Compostela, Spain; 12 Digestive Endoscopy Unit, Agostino Gemelli University Hospital, Rome, Italy; 13 Department of Gastroenterology, Portuguese Oncology Institute of Porto, Porto, Portugal

## Abstract

**Background:**

Existing proposed classification systems for the Papilla of Vater (PV) suboptimally account for all relevant, encountered PV appearances, are too complex or have not been assessed for intra- or interobserver variability. We proposed a novel endoscopic classification system for PV, determined its inter- and intraobserver rates and used the classification system to assess whether the success and complications of needle-knife fistulotomy (NKF) are influenced by the morphology of the PV.

**Methods:**

The classification system was developed by expert endoscopists. To evaluate the inter- and intraobserver agreement, an online questionnaire was sent to 20 endoscopists from several countries (10 experts and 10 nonexperts) that included 50 images of papillae of Vater divided among various categories. Four weeks later, a second survey, with the images from the first questionnaire randomly reordered, was sent to the same endoscopists. The inter- and intraobserver agreements among the experts and nonexperts was calculated. Using the proposed classification system, all 361 consecutive patients who underwent NKF for biliary access to a naïve papilla were prospectively enrolled in the study.

**Results:**

The novel classification system comprises 7 categories: type I, flat type, lacking an oral protrusion; type IIA, prominent tubular nonpleated type, with an oral protrusion and < 1 transverse fold over the oral protrusion; type IIB, prominent tubular pleated type, with an oral protrusion and > 2 transverse folds over the oral protrusion; type IIC: prominent bulging type, with an enlarged and bulging oral protrusion; type IIIA, diverticular-intradiverticular type, with a papillary orifice inside the diverticulum; type IIIB: diverticular-diverticular border type, with a papillary orifice less than 2 cm from the diverticular border; type IV: unclassified papilla, with no morphology classified in the other categories. The interobserver agreement between experts was substantial (K = 0.611, 95% CI 0.498–0.709) and was higher than that between nonexperts (K = 0.516; 95% CI 0.410–0.636). The intraobserver agreement was substantial among both experts (K = 0,651; 95% CI 0.586–0.715) and nonexperts (K = 0.646, 95% CI 0.615–0.677). In a multivariate model, type IIIA and IIIB were the only independent risk factors for difficult rescue NKF biliary cannulation (*P* = 0.003 and *P* = 0.019, respectively), and type I and type IIB were the only independent risk factors for a prolonged cannulation time using NKF (*P* < 0.001 and *P* = 0.005, respectively).

**Conclusions:**

The novel endoscopic classification system for PV is highly reproducible among experienced ERCPists according to the substantial level of agreement between experts. However, nonexperts require further training in its use. Using the novel classification system, we identified different types of papillae significantly associated with a lower efficacy of NKF and a prolonged time to obtain successful biliary cannulation using NKF.

## Introduction

The success of therapeutic biliary Endoscopic Retrograde Cholangiopancreatography (ERCP) depends on selective biliary cannulation, which is the most important step of the procedure [1, 2]. However, conventional techniques for gaining access into the biliary system may fail in 5–35% of cases [1–4]. Therefore, in the subset of patients that undergoes ERCP, there is a need for more advanced access techniques. Such a rescue strategy includes several procedures, namely, pancreatic guidewire-assisted biliary cannulation and precut biliary sphincterotomy. Several precut modalities are available, and one of these techniques, namely, needle-knife fistulotomy (NKF), is increasing in popularity. Previous studies have suggested that although overall cannulation rates were comparable between both precut techniques, the rate of post-ERCP pancreatitis (PEP) was significantly lower after NKF [5–8]. Therefore, the European Society of Gastrointestinal Endoscopy (ESGE) has recently recommended needle-knife fistulotomy as the preferred technique for precutting [9]. Some authors have suggested that NKF should only be undertaken for papillae with a long intramural segment to avoid adverse events, namely, PEP and perforations [9–11]. Authors have argued that prominent papillae are not only easier to cut but also associated with larger common bile ducts (CBDs), not thin bile ducts, which have been suggested to be an independent risk factor for complications in patients subjected to rescue precut techniques [2, 12, 13]. However, a recent study has shown that the morphology of the papilla must not be used as a predictor of the diameter of the terminal CBD (t-CBD), as there is no correlation between these two items [14]. Therefore, it is important to know if the macroscopic appearance of the major duodenal papilla influences the success of NKF and if endoscopists decision in undertaking NKF should be based on the morphology of the ampulla of Vater. Until now, no studies have addressed whether the success and complications of NKF are influenced by the endoscopic morphology of the major papilla.

The eponym "papilla of Vater" is derived from Abraham Vater, a German anatomist who first published a description of the papilla in 1720 [15]. In his original work, Vater recognized distinct variations in the anatomy of the ampulla among individuals [16]. The widespread use of high-quality digital images has led to various reports on the morphology of the major papilla, and there is a clear need and desire for an endoscopic classification system based on the morphology of the ampulla. During the last decade, several authors have proposed different classification systems and have used them to predict the success of cannulation, the rate of complications and the need for more advanced access techniques [10, 17–20]. However, only one endoscopic classification system has been subjected to an inter- and intraobserver agreement study. Haraldsson et al. proposed an endoscopic classification system of 4 types of papillae [18]. Two years later, they reported, in a subsequent study, that the classification system was useful in predicting difficult biliary cannulation not only for experienced endoscopists but also for beginners [19]. However, other distinct morphological features were not taken into account in this classification system, as suggested in other reports [9, 17, 20]. Therefore, existing proposed classification systems suboptimally account for all relevant encountered papilla of Vater appearances, are too complex or have not been assessed for inter- or intraobserver variability. We conducted a study aiming to (a) devise a novel extended classification system for the papilla of Vater and assess the inter- and intraobserver agreement rates among endoscopists with different levels of expertise (expert versus nonexpert endoscopists) and (b) assess whether the success and complications of NKF are influenced by the morphology of the ampulla of Vater using our novel extended classification system of the major papilla.

## Methods

### Study 1: A novel extended classification system for the ampulla of Vater

#### Type of study, patients and endoscopists

This study was divided into two parts: part 1, in which the classification system was developed, and part 2, in which the reproducibility of the novel extended classification system was evaluated.

The study population comprised all patients with a naïve papilla who were referred for ERCP at Hospital Santa Luzia in Viana do Castelo, a hospital affiliated with the University of Minho. Under full duodenal inflation, a complete set of at least four pictures was taken and stored in a digital database. This study was conducted in compliance with the International Conference on Harmonization guidelines for Good Clinical Practice (E6) and the 2013 Declaration of Helsinki. All enrolled patients provided informed written consent before their procedures, and the Ethics Committee at our institution approved this study (ULSAM 47/2018-10/10).

#### Part 1: development of the classification system

The classification system was developed by expert endoscopists during a 6-month period, using a modified Delphi method to achieve consensus [21]. First, a set of 550 still images from our papilla major library database (all available images) were distributed among and reviewed by all endoscopists involved in part 1. After a first meeting in which the main morphologic criteria were agreed upon, subsequent meetings took place to complete the classification system. The proposed morphologic criteria used in this classification system were (1) the shape of the papilla, (2) the protrusion of the oral segment of the papilla which was defined as the aspect of the intra-duodenal portion of the ampullary complex and its 'protrusion' into the duodenal lumen, (3) the number of transverse folds on the oral portion of the papilla, (4) the presence of a diverticulum, regardless of the oral protrusion of the ampullary complex and (5) the inclusion of a category named unclassified papilla to accommodate the remaining papillae. During the process of achieving consensus the shape of the papillary orifice and the ratio between the longitudinal measure versus the transverse measure of the papilla were consensually not included in the classification because it would transform the classification in a very complex system and therefore not suitable for routine clinical practice. A web-based platform was used for structured discussion and voting to refine the proposal. The final version of the classification system (Viana Classification) was voted on a working meeting held in the city of Viana do Castelo, on the northern coast of Portugal.

#### Part 2: reliability of the classification system

For the assessment of the inter- and intraobserver agreement rates among the endoscopists, we formed 2 groups: endoscopists with expertise in ERCP (n = 10) from different countries (Portugal, Belgium, Italy, Spain and the Netherlands) and a group of gastroenterology residents with limited experience in ERCP (less than one year of experience and less than 200 ERCPs performed/monitored), required during the residency curriculum (n = 10; all Portuguese). Expertise with ERCP was defined as having performed more than 1000 ERCPs and having more than 6 years of experience. The endoscopists involved in part 1 did not participate in the agreement study. The reliability of the classification system was assessed using a web-based questionnaire with 50 still papilla images selected prospectively from our library after being classified. The images were randomly selected.

The survey started with a description of the classification system and some training questions followed by direct feedback for the respondents to become familiar with the classification system. Each question in the survey was accompanied by a large still image of an ampulla and presented on a single page, always with an explanatory picture of the classification system at the bottom. Respondents needed to make an obligatory choice from a dropdown box to identify the particular type, from the seven possible types, to which they categorized each image. All the participants had unlimited time to view each papilla or the complete set of papillae. Furthermore, they were allowed to return and change a previous decision on the type of papilla selected.

The questionnaire was distributed to 20 endoscopists (see above). Data from each endoscopist, in addition to their classification as experienced/trainee, were anonymized and entered directly into a database for later analysis. The participants were not able to record or keep a register of the answers from the first survey. One month after the completion of the first questionnaire, a second version of the survey was sent to the same endoscopists to evaluate intraobserver agreement. This second version was identical to the first version, except that the images were randomly reordered.

### Study 2: Influence of the novel extended classification system on the outcomes of NKF

This was an observational multicenter study. Between May 2018 and October 2020, all consecutive patients who underwent NKF for biliary access to a naïve papilla were enrolled in the study and were followed prospectively (see below for endoscopists involved). The exclusion criteria were as follows: (1) patients with surgically altered anatomy, (2) patients with tumors of the papilla and (3) patients unable to be positioned prone or supine for cholangiography acquisition.

The collected data included patient demographics, indication for ERCP, underlying biliary pathology, therapeutic interventions, endoscopic morphology of the major papilla, length of time needed to achieve biliary cannulation after starting NKF, intraprocedure complications and postprocedure complications. This study was conducted at 3 institutions with a total annual load of 1300 ERCPs. This study was conducted in compliance with the International Conference on Harmonization guidelines for Good Clinical Practice (E6) and the 2013 Declaration of Helsinki. All of the patients provided informed written consent before their procedures. The Ethics Committee at each institution approved this observational study (EDOC/ULSBA/15,191, ULSAM 42/2019 and EC/59-2018).

#### Endpoints and definitions for the study 2

The primary endpoint was the influence of the endoscopic appearance of the major papilla on the success of NKF and adverse events. The secondary endpoint included the influence of the morphology of the ampulla on the time to achieve biliary cannulation and the technical success rate of NKF globally and at initial ERCP.

The t-CBD diameter was assessed 1 cm above the distal end of the CBD on cholangiography, as described elsewhere [14]. Biliary pathology was divided into benign and malignant. The time needed to achieve biliary cannulation using NKF was defined as the length of time between the first contact with the papilla by a needle-knife and visualization of a guidewire into the biliary duct. The adverse event rate was defined as the rate of procedure-related adverse events. Overall adverse events, namely, post-ERCP pancreatitis (PEP), ERCP bleeding and retroperitoneal perforation, were classified and graded according to consensus guidelines [7, 22, 23].

#### Intervention, endoscopists and PEP prevention

ERCP procedures were performed with patients in the prone position under sedation with propofol administered by an anesthesiologist. Before starting biliary cannulation, at least four still images of the papilla were taken at full inflation and stored in a digital database. The classification system was provided during the ERCP; retrospective classification of the papilla using videos or still images after ERCP was not possible. Patients without a classified papilla at initial ERCP were excluded. The endoscopists performing NKF in the study (JC, LL) have an annual load above 500 ERCPs/year and have achieved selective biliary cannulation in more than 80% of patients using standard access techniques. Both have performed more than 800 NKFs in their career and more than 80 NKFs/year in the last 5 years. In the study period, NKF was performed early, which was defined as an NKF attempt after 5 min of unsuccessful attempts to achieve deep biliary cannulation using standard methods. The NFK technique has been described extensively elsewhere [1–4, 6, 9, 17, 24, 25].

All patients underwent routine rectal administration of 100 mg of diclofenac or indomethacin immediately before ERCP. In all cases involving pancreatic opacification or guidewire passage into the pancreatic duct, prophylactic pancreatic stenting was performed according to known guidelines [9, 26].

### Statistical analysis

#### Study 1: A novel extended classification system for the ampulla of Vater

The inter- and intraobserver agreement was calculated for the entire group, and a subanalysis was subsequently performed by dividing the endoscopists into two groups according to their experience. Intraobserver agreement was calculated by comparing the answers from the same endoscopist from the two surveys. The degree of agreement between the answers of the endoscopists was assessed using Fleiss' kappa statistics for unordered categories as follows: k between 0.01 and 0.2, slight; K between 0.21 and 0.4, fair; K between 0.41 and 0.6, moderate; K between 0.61 and 0.8, substantial; and K between 0.81 and 1.0, almost perfect agreement [27]. The proportion of agreement with the reference, the predefined classification system, was evaluated using relative frequencies against the observed responses by the 20 endoscopists surveyed.

#### Study 2: Influence of the novel extended classification system on the outcomes of NKF

Qualitative variables are summarized using absolute and relative frequencies, and quantitative variables are summarized using the mean and standard deviation or the median and range, depending on their distribution profiles. The normality of the quantitative variables was assessed using a histogram of the distribution of the variables.

Differences between categorical variables were tested using the chi-square test and Fisher´s exact test.

To explain successful biliary cannulation in the initial ERCP, qualitative binary regression models with logit specification were defined. The average marginal effects, standard errors and individual significance tests are presented in tables. To explain the overall cannulation time, a multiple linear regression model was defined using ordinary least squares (OLS) to estimate the coefficients. In the multivariate model, distal malignant stricture was one of the variables used in the analysis.

The null hypothesis was rejected when the test statistic p-values were less than < 0.05. Statistical analysis was performed and graphics were generated using Stata software (StataCorp. 2015. Stata Statistical Software: Release 14. College Station, TX: StataCorp LP).

## Results

### Study 1: A novel extended classification system for the ampulla of Vater

#### The classification system

**Fig. 1:**
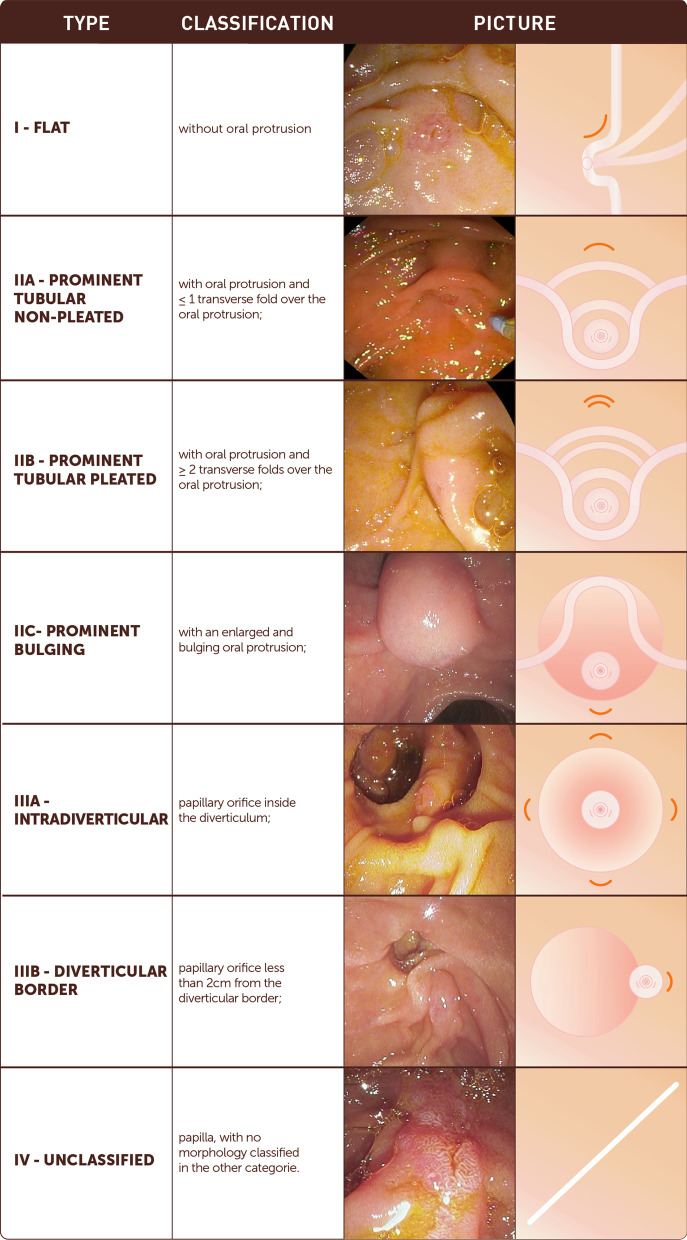
Novel classification of the endoscopic appearance of the papilla of Vater

The proposed classification system includes 7 categories: type I: flat type, without an oral protrusion; type IIA: prominent tubular nonpleated type, with an oral protrusion and < 1 transverse fold over the oral protrusion; type IIB: prominent tubular pleated type, with an oral protrusion and > 2 transverse folds over the oral protrusion; type IIC: prominent bulging type, with an enlarged and bulging oral protrusion; type IIIA: diverticular-intradiverticular type, with a papillary orifice inside the diverticulum; type IIIB, diverticular-diverticular border type, with a papillary orifice less than 2 cm from the diverticular border; type IV: unclassified papilla, a type of papilla with no morphology classified into the other categories (Fig. 1).

#### Reliability

All questionnaires were completed by the 20 endoscopists. The questionnaire contained examples of 8 type I papillae, 8 type IIA papillae, 8 type IIB papillae, 8 type IIC papillae, 6 type IIIA papillae, 6 type IIIB papillae and 6 type IV papillae.

The overall interobserver agreement was moderate (K = 0.567) (Table 1). When subanalysis was performed according to group, the level of agreement was substantial for the experts (K = 0.611) and moderate for the trainees (K = 0.516). The overall intraobserver agreement was substantial for both the experts (K = 0.651) and nonexperts (K = 0.646).

**Table 1:** Interobserver and intraobserver agreement among experts and nonexperts

Intraobserver agreement	Κ (95% CI)	Agreement
All endoscopists	0.648 (0.641–0.656)	Substantial
Experts	0.611 (0.498–0.709)	Substantial
Trainees	0.516 (0.410–0.636)	Substantial
*Interbserver agreement*		
All endoscopists	0.567 (0.470–0.675)	Moderate
Experts	0.651 (0.586–0.715)	Substantial
Trainees	0.646 (0.615–0.677)	Moderate

**Fig. 2:**
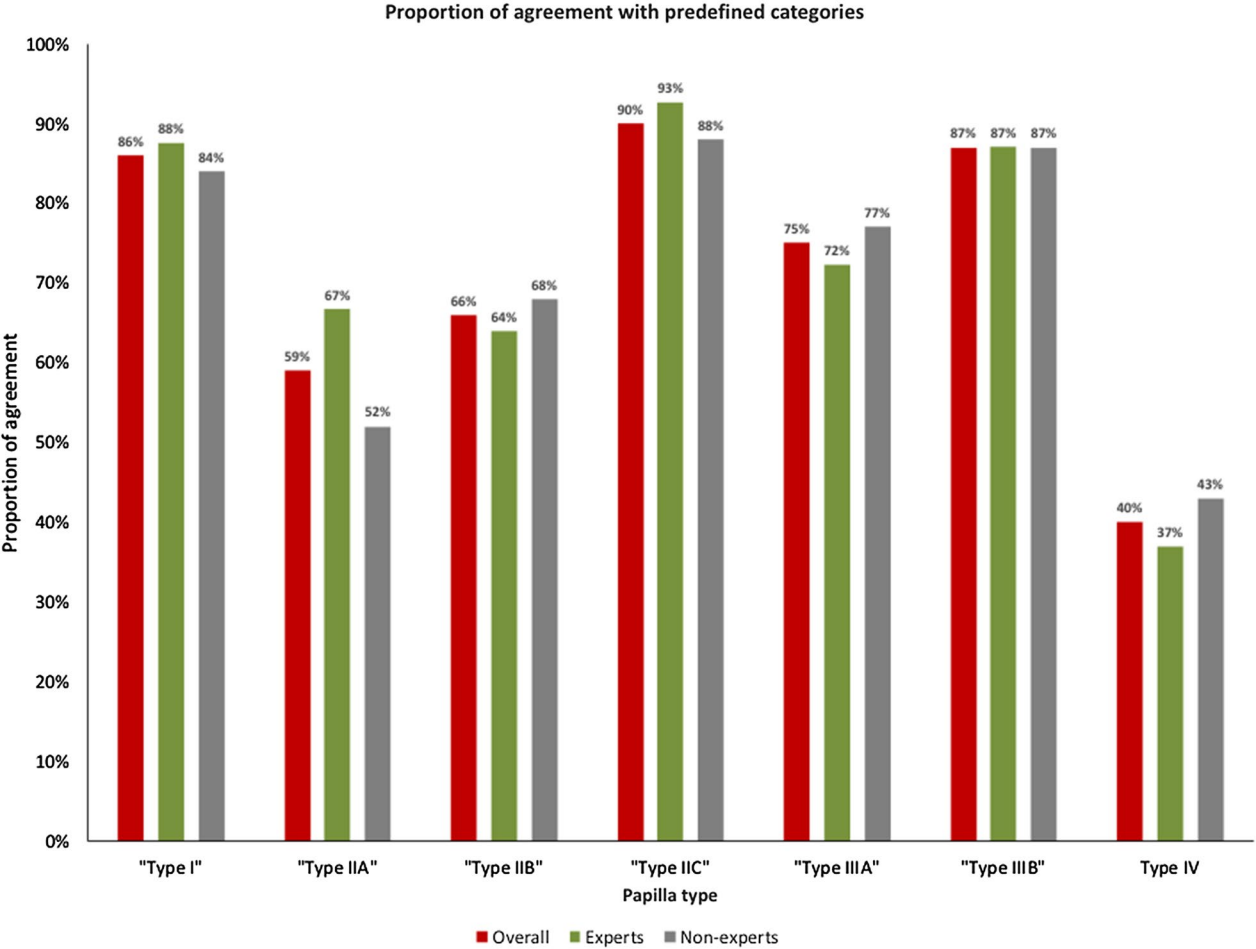
Proportion of agreement between the reference (predefined papilla): overall and among experts and nonexperts

The rate of agreement among the 20 endoscopists was 71.85% and varied according to papilla type (range 40–90%) (Fig. 2). Type I papillae (flat type), type IIC papillae (bulging type) and type IIIA papillae (intradiverticular type) had a high level of concordance. The level of agreement was lower for type IIA (prominent nonpleated type) and type IIB (prominent pleated type) papillae, with 67% and 64% concordance, respectively. The category with the least agreement was the unclassified papilla (type IV) with 40% concordance. The rate of agreement between experts and nonexperts was similar except for type IIA (prominent tubular nonpleated type) papillae, in which the rate of agreement between experts and trainees was substantially different (52% vs 67%).

A more detailed examination of the distribution of answers of the endoscopists revealed that there was an interchangeability of choices between type IIA and Type IIB, and most of the endoscopists that did not consider type IV instead selected type I in such cases (Table 2).

**Table 2:** Proportion of agreement between the reference (predefined papilla) and the survey responses for each type of papilla

Classification proposed	Survey responses by the 20 endoscopists						
	Type I (%)	Type IIA (%)	Type IIB (%)	Type IIC (%)	Type IIIA (%)	Type IIIB (%)	Type IV (%)
Type I	86	2.5	1.5	0	0	0	12
Type IIA	4	59	31	4	0	0	2
Type IIB	0	24	66	10	0	0	0
Type IIC	0	0	9	90	0	0	1
Type IIIA	3.5	0	0	0	75	15	6.5
Type IIIB	0.5	8	2	0	2	87	0.5
Type IV	47	0	2	0	9	2	40

### Study 2: Influence of the novel extended classification system on the outcomes of NKF

#### Patients

**Fig. 3:**
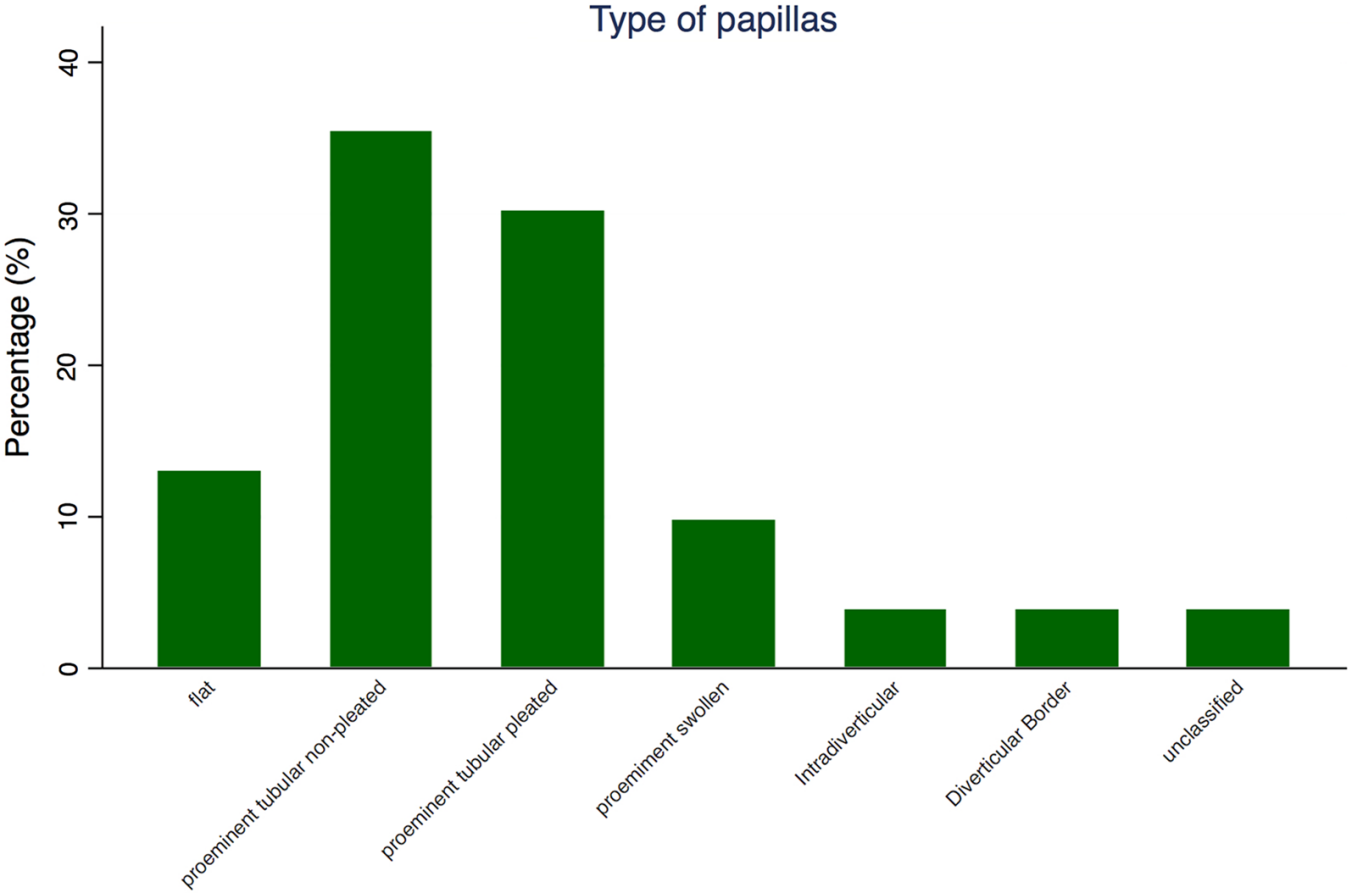
Distribution of the different types of papillae among 361 patients

During the study period, namely, 2639 naïve papillae were subjected to ERCP. Of these patients, 361 underwent NKF as a rescue method for biliary cannulation early, namely, after five minutes of biliary cannulation attempts using standard techniques. Therefore, 361 patients (156 males and 205 females), with a mean age of 69.6 years (range 18–97 years), were enrolled in the study. Classification of the papilla into the predefined types was possible for all patients. The distribution of the different papillae is shown in Fig. 3 and Table 3. Type IIA (prominent tubular nonpleated type) was the most frequent (128 patients, 35.4%), followed by type IIB (prominent tubular pleated type), which was found in 109 patients (30.2%). Patient demographics, group distribution and group characteristics are summarized in Table 3.

**Table 3:** Patient’s demographics, group distribution and group characteristics of 361 patients submitted to NKF

	Type I n (%) 47 (13.0)	Type IIa n (%) 128 (35.5)	Type IIb n (%) 109 (30.2)	Type IIc n (%) 35 (9.6)	Type IIIa n (%) 14 (3.9)	Type IIIb n (%) 14 (3.9)	Type IV n (%) 14 (3.9)	*p*
Age, median	74	70	72	74	79	80	75	0.074
Sex, n (%)								0.528
Male	24 (51)	55 (43)	49 (45)	15 (43)	4 (29)	3 (21)	6 (43)	
Female	23 (49)	73 (57)	60 (55)	20 (57)	10 (71)	11 (79)	8 (57)	
Final diagnosis, n (%)								
CBD stones	23 (48.9)	83 (64.9)	51 (46.8)	20 (57.1)	11 (78.6)	7 (50.0)	3 (21.4)	0.010
Malignant stricture	16 (34.0)	22 (17.2)	26 (23.9)	7 (20)	1 (7.1)	1 (7.1)	5 (35.7)	0.229
Leaks	1 (2.1)	3 (2.3)	5 (4.6)	1 (2.9)	0	0	0	0.935
Other findings	7 (15.0)	20 (15.6)	27 (24.8)	7 (20.0)	2 (14.3)	6 (42.9)	6 (42.9)	0.356

NKF, needle knife fistulotomy; CBD, common bile duct

#### Influence of the endoscopic appearance of the major papilla on the success of NKF and adverse events

Overall, regarding the initial cannulation rate, selective biliary cannulation was obtained in 334/361 (92.5%) of the patients. The initial cannulation rate using NKF was significantly different between the different types of papillae (Table 4 and Fig. 2). Type IIB (prominent tubular pleated type), type IIIA (diverticular-intradiverticular type) and type IIIB (diverticular-diverticular border type) were associated with the lowest cannulation rates using NKF. In the multivariate model (Table 5), type IIIA and IIIB were the only independent risk factors for difficult biliary cannulation (odds ratio: 0,567; 95% confidence interval 0.008–0384, *P* = 0.003 and odds ratio: 0,081; 95% confidence interval 0.009–0.656, *P* = 0.019, respectively). Overall biliary cannulation was obtained at the second attempt in 351/361 patients (97.22%). There were no significant differences in the cannulation rate between the different types of papillae when using NKF at the second attempt.

**Table 4:** Influence of the endoscopic appearance of the major papilla on the success of NKF and adverse events in 361 patients

	Type I n (%) 47 (13.0)	Type IIa n (%) 128 (35.5)	Type IIb n (%) 109 (30.2)	Type IIc n (%) 35 (9.6)	Type IIIa n (%) 14 (3.9)	Type IIIb n (%) 14 (3.9)	Type IV n (%) 14 (3.9)	*p*
Biliary cannulation, n (%)								
Success in first ERCP	44 (93.6)	124 (96.9)	100 (91.7)	35 (100)	9 (64.3)	10 (71.4)	12 (85.7)	0.000
Overall biliary cannulation	46 (97.9)	127 (99.2)	105 (96.3)	35 (100)	13 (92.9)	12 (85.7)	13 (92.9)	0.041
Cannulation time (mins), median (p25–p75)	14.3 (7–26)	5.0 (1–38)	8.8 (7–15)	5.5 (3–12)	8.0 (7–10)	9.5 (5–14)	8.7 (5–21)	0.0001
*Adverse events, n (%)*								
Overal	5 (10.6)	7 (5.5)	10 (9.2)	3 (8.6)	0	2 (14.3)	0	0.549
Pancreatitis	4 (8.5)	5 (3.9)	5 (4.6)	2 (5.7)	0	2 (14.3)	0	0.488
Bleeding	1 (2.1)	2 (1.6)	5 (4.6)	1 (2.9)	0	0	0	0.837
Perforation	0	0	0	0	0	0	0	–

NKF, needle knife fistulotomy

**Table 5:** Results of a multivariate logistic regression model to evaluate predictors of successful biliary cannulation in 361 observations

	OR	*p*	95% CI	
			Lower	Upper
Sex	1.903	0.2330	− 0.521	9.949
Age	1.009	0.668	0.967	1.053
Viana classification				
I	1.321	0.361	0.109	2.235
IIa	0			
IIb	0.495	0.361	0.109	2.235
IIc	1.103	0.458	1.002	2.236
IIIa	0.567	0.003*	0.008	0.384
IIIb	0.081	0.019*	0.009	0.656
IV	0.981	0.582	0.123	1.026
Biliary stenosis	0.633	0.489	0.173	2.307
CBD diameter	1.090	0.204	0.953	1.247

*p < 0.01

The cannulation time using NKF was significantly different among the different types of papillae, with type I, type IIB and type IIIB being associated with the longest cannulation times when using NKF. However, in the multivariate regression model (Table 6), type I and type IIB were the only independent risk factors for a prolonged cannulation time using NKF (odds ratio: 8,266; 95% confidence interval 4,077–12.453, *P* < 0.001 and odds ratio: 3.593; 95% confidence interval 1.114–6.071, *P* = 0.005, respectively).

**Table 6:** Results of a multiple regression model to evaluate predictors of cannulation time

	Coef	*p*	95% CI	
			Lower	Upper
Sex	1.326	0.208	− 0.742	3.394
Age	0.031	0.234	− 0.020	0.082
Viana classification				
I	8.266	0.000*	4.077	12.453
IIa	0			
IIb	3.593	0.005*	1.114	6.071
IIc	1.237	0.466	− 2.100	4.573
IIIa	2.235	0.327	− 2.245	6.715
IIIb	1.157	0.468	− 1.979	4.294
IV	8.331	0.072	− 0.747	17.411
Biliary stenosis	2.591	0.063	1.960	3.220
CBD diameter	− 0.143	0.319	− 0.426	0.139

*p < 0.01; R squared = 0186

In total, procedure-related adverse events (Table 4) were observed in 27/361 of the patients (7.5%). The rate of adverse events was not significantly different among the different types of papillae. Overall, the most common adverse event, pancreatitis, was observed in 18/361 of the patients (4.9%).

## Discussion

In this study, a novel extended endoscopic classification system of the major papilla was proposed. The interobserver agreement was substantial among the expert participants and moderate among the group of nonexperienced endoscopists. The results were highly reproducible by all endoscopists based on the substantial intraobserver agreement among the expert and nonexpert participants. Furthermore, using the new classification system, we identified different types of papillae significantly associated with a lower efficacy of NKF and a prolonged time to obtain successful biliary cannulation using NKF.

Previous studies have been undertaken to devise a classification system for the major papilla. In 2007, Horiuchi et al. classified the major papilla based on the size of the oral protrusion into the duodenal lumen into small, large or swollen [9]. The authors suggested that this classification system could be used to guide the type of precut sphincterotomy performed for patients with a difficult biliary cannulation. The authors concluded that NKF had the highest success rate when performed for swollen papillae, which is similar to our type IIC. However, this classification system was not validated, and the definition of a difficult cannulation as used in that study was arbitrary and has not been used by most other authors or guidelines. Several years later, Lee et al. described four types of papillae: nonprominent, prominent, bulging and a fourth type called the distorted type [17]. In their original report, the authors used this classification system to evaluate the success and safety of precut fistulotomy for difficult biliary cannulation as performed by endoscopists with a low level of training. The authors reported that the success of NKF was similar between prominent and nonprominent papillae, but bulging papillae had a high rate of success of biliary cannulation using NKF (96.8%). However, this classification system did not go through a validation process, and the definition used for a difficult biliary cannulation was a personal definition that is not used in other reports or guidelines on the subject. In short, the first two attempts to classify the major papilla used the morphological characteristics of the ampulla to guide the success of NKF. Therefore, it is important to know if a novel classification of the major duodenal papilla influences the decision to undertake NKF and if it is associated with adverse events. In 2016, a Scandinavian group proposed a new classification system, reporting 4 types of papillae: type 1, regular; type 2, small; type 3, protruding or pendulous; and type 4, creased or rigid [18]. This classification system was evaluated with an inter- and intraobserver agreement study among 18 endoscopists (nine experts and nine nonexperts) using a set of 50 still images. The overall interobserver agreement was substantial (K = 0.62) and similar between the experts (K = 0.63) and nonexperts (K = 0.61). Furthermore, the intraobserver agreement was also substantial (K = 0.66) and was again similar between the experts (K = 0.68) and nonexperts (K = 0.62). Two years later, the same group reported the relevance of using this classification system to assess the difficulty of bile duct cannulation [19]. They reported that type 2 and type 3 papillae were more frequently difficult to cannulate and that cannulation might even fail more frequently if a beginner starts it. However, despite the merit of this work, this classification system seemed incomplete immediately and did not recognize other morphological forms. Therefore, in 2019, a group from Japan proposed a novel classification system based on (1) the ratio of the length between the oral protrusion and the transverse diameter of the papilla (protrusion pattern) and (2) the papilla pattern, which indicates the surface pattern of the papilla, including the morphology of the orifice [20]. They proposed 8 types of papillae based on the ratio of the length of oral protrusion to the transverse diameter of the papilla, with an additional 5 types of papilla patterns. They subjected this proposed classification system to an internal validation method in which 3 experienced endoscopists agreed on the classification system. The manuscript did not specify if this validation was performed using still images and if the evaluators were the same as the original authors of the classification system, creating a large bias immediately in terms of the validation. Using this classification system, the authors reported that papillae with large protrusions represented an independent risk factor for a difficult cannulation. Despite the merits and efforts of the authors, this classification system was not tested for inter- and intraobserver agreement, and the classification system is of such complexity that it does not seem suitable for routine clinical practice. At present, the Nordic classification system is the only one suitable for use in routine practice. However, this system may have missed relevant macroscopic appearances of the ampulla of Vater, and there are several limitations that should be taken into account. Type 3 in the Nordic classification system includes all protruding papillae and does not include a necessary division between different types of protrusions into the lumen. Protruding papillae can assume different forms, namely, with several pleats and bulging. Therefore, we believe that prominent papillae should be divided into 3 groups: nonpleated, pleated and bulging, which intuitively are associated with distinct and different difficulties of cannulation when using not only standard techniques but also distinct advanced assessment techniques, potentially leading to differential risks and types of complications. All experienced endoscopists known from practice that prominent papillae with several folds do not represent an easy task for cannulation, especially without the use of any advanced access techniques. Papillae with more pleats (Type IIB) tend to have a longer intraduodenal portion of the common bile duct, several pleats and most of the time the orifice is hanging down into the duodenal lumen. Many times, these papillae are under overlying folds and deep cannulation is not generally easy. All experimented endoscopists have intuitively the idea that these papillae could be challenging. In current study Type IIB was associated with the lower levels of deep biliary cannulation (only Type III-the diverticular papillae performed poorly) and furthermore Type IIB was in independent risk factor for prolonged cannulation type. In future studies which are on their way and are the next step we will evaluate the performance of Type IIB when using traditional methods of cannulation. Recently, Adler [35] remembered that papillae under multiple draping, overlying folds (sometimes humorously referred to as the Shar-Pei dog papilla) should be taken into account in a classification system (our tubular pleated type). Additionally, true bulging papillae are challenging to cannulate with standard techniques and are probably better cannulated by precut techniques, namely, needle-knife suprapapillary fistulotomy [7, 17, 28–31]. Furthermore, the Nordic classification system does not include diverticular papillae, which have been associated with difficult cannulation and may be different from intradiverticular or papillae located on the edge (border) of the diverticulum. Most of the ERCPists report that ERCP for intradiverticular papillae continues to be a challenge [32–34], but no large series have yet to prove this statement, and it is clear that a new classification system that objectifies cannulation difficulty should include this type of papillae as strongly suggested by a recent paper from a well-known ERCPist [35]. Another possible conflicting issue with the work of Haraldsson et al. is their type 1 papilla (regular), which has no clear definition and therefore is not easy to classify during endoscopic visualization. Instead of regular papilla, we included a type IV papilla to include all possible variations in papillary morphologies, which we named unclassified papilla to accommodate other, less frequent endoscopic appearances of the ampulla and those that are impossible to classify. Finally, and accordingly with previous classification systems, small papillae (our flat type) have been associated with difficult cannulation and complications and should be included in any classification system of the major papilla [7, 10]. Therefore, and due to the limited morphological appearances available in the classification system from Haraldsson et al., we proposed a novel, extended classification system that accommodates an extended number of distinct features related to the endoscopic appearance of the papilla of Vater. Not surprisingly, a recent editorial suggested that classification systems with more types were strongly needed [35].

In this study, trainees had a poorer intraobserver agreement than experienced ERCPists, suggesting that papilla evaluation is easier for endoscopists who are used to examine papillae in daily practice. We observed a high level of concordance for the flat type, bulging type and diverticular type, suggesting that these types of papillae are easy to learn and differentiate. However, prominent papillae were more difficult to classify, namely, the difference between pleated and nonpleated papillae, due to the nature and number of pleats, and this difficulty was more pronounced for the nonexpert group. In fact, this was the only major difference between the answers of the experts versus nonexperts and the main reason why the interobserver rate was poorer in the trainee group. We believe that with training, endoscopists will be able to differentiate easily between the two types of papillae. Furthermore, we intuitively believe that these two types of papillae will behave differently in terms of cannulation difficulty, complications and precut techniques. Finally, the category of unclassified papilla had the poorest results with a low level of agreement. This may have resulted from the need of the endoscopist to classify an unexpected morphology into something else that was more common, namely, flat papillae, which more frequently can be confounded with the unclassified papilla category.

In this study, it is clear that flat papillae and all types of prominent papillae can be subjected to NKF with a success above 90% when performed by experienced endoscopists. Furthermore, diverticular papillae were the only independent risk factor for using NKF, and it is clear that this type of papillae should be addressed with a novel classification. Interestingly, diverticular papillae were not associated with a significantly longer duration for performing a successful NKF. This observation was associated with flat and tubular pleated papillae. These two types of papillae, one small and the other with a long intraduodenal portion of the common bile duct under multiple folds, are challenging to treat with NKF and therefore are associated with long times to achieve successful biliary cannulation.

Future studies will hopefully prove whether this classification system is easy to use in daily practice and consistently reproduced by all endoscopists. Furthermore, future studies should address the utility of this classification system in predicting the difficulty of cannulation using conventional techniques, complications, and which types of papillae are most suitable in each differential stage of the training process for novice ERCPists.

The present study has several limitations. First, we used still images instead of video sequences, which may only represent part of the morphological appearance of the ampulla. Another potential limitation is the use of Fleiss' kappa statistic, which while appropriate for testing whether agreement exceeds chance, has some limitations, as it makes no distinction among various types and sources of disagreement. Because it is affected by prevalence, it may not be appropriate to compare kappa between different studies or populations. Nonetheless, kappa can provide more information than a simple calculation of the raw proportion of agreement [36].

The strengths of our study include the extension of previous classification systems, attempting to accommodate a larger number of papilla morphologies that were not considered in previous studies, the number of endoscopists included and the inclusion of experts from different countries. Furthermore, we used this novel classification system to observe that different types of papillae were associated with different success rates of biliary cannulation and different times for successfully performing NKF using the largest series of NKF ever reported.

## Conclusion

Our novel classification system for appraising the endoscopic appearance of the papilla of Vater is highly reproducible among experienced ERCPists based on a substantial level of agreement between experts. Although the results of the survey were consistently reproduced by all endoscopists, nonexperts will require further training in its use. Using this novel classification system, we observed that intradiverticular papillae were independent risk factors for difficult cannulation using NKF and flat and tubular pleated papillae were independent risk factors for a prolonged cannulation time using NKF. Future studies will need to demonstrate whether this extended novel classification system has clinical utility in terms of actually being able to prospectively predict cannulation difficulty with standard techniques and complication risks. Furthermore, this novel classification system would be a guide to selecting the most optimal biliary cannulation option for each patient though the subsequent clinical verification process.

## Data Availability

The datasets generated and/or analyzed in the present study are available from the corresponding author on reasonable request.

## Abbreviations

PV: Papilla of Vater
NKF: Needle-knife fistulotomy
ERCP: Endoscopic retrograde cholangiopancreatography
ESGE: European Society of Gastrointestinal Endoscopy
PEP: Post-ERCP pancreatitis
t-CBD: Diameter of the terminal CBD
